# Microcirculation and Mitochondria: The Critical Unit

**DOI:** 10.3390/jcm12206453

**Published:** 2023-10-11

**Authors:** Guangjian Wang, Hui Lian, Hongmin Zhang, Xiaoting Wang

**Affiliations:** 1 Department of Critical Care Medicine, Peking Union Medical College Hospital, Peking Union Medical College, Chinese Academy of Medical Sciences, Beijing 100730, China; wgjdoctor@student.pumc.edu.cn (G.W.); zhanghongmin1@pumch.cn (H.Z.); 2 Department of Health Care, Peking Union Medical College Hospital, Peking Union Medical College, Chinese Academy of Medical Sciences, Beijing 100730, China; lianhui@pumch.cn

**Keywords:** critical illness, microcirculation, mitochondria, critical unit, monitoring technique, treatment strategy

## Abstract

Critical illness is often accompanied by a hemodynamic imbalance between macrocirculation and microcirculation, as well as mitochondrial dysfunction. Microcirculatory disorders lead to abnormalities in the supply of oxygen to tissue cells, while mitochondrial dysfunction leads to abnormal energy metabolism and impaired tissue oxygen utilization, making these conditions important pathogenic factors of critical illness. At the same time, there is a close relationship between the microcirculation and mitochondria. We introduce here the concept of a “critical unit”, with two core components: microcirculation, which mainly comprises the microvascular network and endothelial cells, especially the endothelial glycocalyx; and mitochondria, which are mainly involved in energy metabolism but perform other non-negligible functions. This review also introduces several techniques and devices that can be utilized for the real-time synchronous monitoring of the microcirculation and mitochondria, and thus critical unit monitoring. Finally, we put forward the concepts and strategies of critical unit-guided treatment.

## 1. Introduction

Regarding patients with acute stroke, neurologists often say, “time is brain” or “time is neuron”. Then, what should intensive care physicians pay attention to when dealing with critically ill patients? With the in-depth study and understanding of critical illness, the answer to this question has gradually become clear and specific. Critical illness is often accompanied by a hemodynamic imbalance between macrocirculation and microcirculation, as well as mitochondrial dysfunction, both of which are directly related to organ dysfunction and poor prognosis [1]. Microcirculation dysfunction can lead to the inability to ensure adequate oxygen transport to parenchymal cells, and mitochondrial damage leads to abnormal energy metabolism, resulting in oxygen utilization disorders in tissues and cells. As confirmed in septic shock, there is a significant correlation between patient prognosis and adenosine triphosphate (ATP) levels, highlighting the importance of cellular energy metabolism [2]. Therefore, it is not surprising to find that microcirculation and mitochondria may be the most important pathogenic factors of critical illness. Indeed, critical illness must result in microcirculation and mitochondrial dysfunction, which also cause or promote the occurrence and development of critical illness. Consequently, we integrated microcirculation and mitochondria into a concept called the “critical unit”, which we propose is the key link in the occurrence and development of critical illness. On the basis of this understanding, the treatment of critical illness has gradually stepped onto the stage of microcirculation-guided resuscitation and even mitochondria-guided resuscitation; that is, we have arrived at the era of critical unit-guided treatment [3].

## 2. The Key Role of Microcirculation/Endothelium in Critical Illness

### 2.1. Critical Illness—Microcirculation

Microcirculation encompasses a vascular network that is less than 150 mm in diameter and is composed of arterioles, capillary networks, and venules. It is important for transporting oxygen, neutral molecules, and immune cells; removing carbon dioxide and metabolic waste; and maintaining the fluid balance between tissues [4], and it plays a significant role in preserving and recovering organ function in critically ill patients. Microcirculation dysfunction exists in almost all critically ill patients, and the severity is significantly related to organ dysfunction [1]. De Backer et al. [5] demonstrated that sublingual microcirculation in sepsis patients was altered compared with that in healthy subjects.

One of the most important features of microcirculation dysfunction is the heterogeneity of regional perfusion within a few microns of proximity [6]. Tissue hypoxia does not necessarily improve under conditions of normal or higher oxygen transport [7]. The root cause of this phenomenon may be insufficient oxygen uptake due to microcirculatory perfusion dysfunction rather than inadequate systemic oxygen transport, i.e., reduced oxygen uptake caused by functional shunting in microcirculation [7]. Disturbances in microcirculation in critical illness can persist even with improved macrocirculation perfusion, confirming a loss of hemodynamic consistency between macrocirculation and microcirculation. In such cases, microcirculation measurements are of great value in evaluating the effectiveness of the treatment of critical illness. Indeed, microcirculation-guided treatment may improve the prognosis of critically ill patients.

### 2.2. Critical Illness—Endothelium

Endothelial cells are the most important functional cells in microcirculation. Endothelial cells exist in all blood vessels, which mediate and regulate many important physiological functions of the microcirculation, including perfusion, permeability, local coagulation, and immune response [8]. It has been confirmed that endothelial cell damage is the main pathogenic factor of microcirculatory dysfunction in critically ill patients. Endothelial cell death and barrier injury are important pathogeneses of many inflammatory diseases, including acute lung injury (ALI) and myocardial ischemia-reperfusion injury [9]. Therefore, preserving endothelial function should also be a goal of resuscitation. Endothelial dysfunction includes impairments in endothelium-dependent vasodilation and glycocalyx, increased reactive oxygen species (ROS), and decreased endothelial barrier integrity [10,11]. Additionally, the functional morphology and heterogeneous distribution of endothelial receptors in microcirculation make some organs more vulnerable to shock than others [7]. For example, in hemorrhagic shock, the heart is more resistant to anemia than other organs, such as the kidney and intestine [12].

The endothelial glycocalyx (EGC) of the surface layer of endothelial cells plays important roles in regulating vascular permeability, leukocyte and platelet adhesion, shear force, and inflammation. EGC damage is considered an early and sensitive marker of endothelial injury [13]. After acute EGC injury, reconstruction takes approximately 5–7 days, and the full restoration of hydrodynamic activity takes even longer. This delay in healing may, to some extent, explain why impaired microcirculatory perfusion persists in critically ill patients, despite resuscitation and the optimization of macrocirculatory hemodynamics [14].

These observations are not limited to animal experiments. Indeed, many clinical studies support the key role of endothelial cell injury in sepsis-induced organ failure. Deng et al. [15] demonstrated that the inflammation induced by bacterial lipopolysaccharide (LPS) is largely due to its toxic effect on endothelial cells. Recent studies have also shown that the intracellular destruction of LPS can lead to endothelial cell apoptosis and the loss of vascular integrity [16]. In sepsis, exogenous pathogen-associated molecular patterns and endogenous damage-associated molecular patterns (DAMPs) activate the endothelium and may damage its structure and function [17]. Pattern recognition receptors drive endothelial cells to transform into pro-inflammatory phenotypes, thus releasing cytokines, chemokines, procoagulant factors, and adhesion molecules. EGC injury and endothelial cell apoptosis increase vascular permeability, resulting in the leakage of vascular fluid and proteins into the interstitial space, tissue edema, and increased oxygen diffusion distance [18]. Therefore, severe and persistent endothelial cell damage may lead to insufficient microcirculatory perfusion, oxygen delivery, and even life-threatening organ failure.

In addition, extensive endothelial cell dysfunction may be an important part of the pathogenesis of coronavirus disease (2019) [19]. Endothelial dysfunction can induce microvascular leakage, inflammatory reactions, a procoagulant state, and abnormal organ perfusion through cytokine release, oxidative stress, coagulation disorders, and immune cell responses, thus participating in organ failure during virus infection.

## 3. The Important Role of Mitochondria in Critical Illness

Mitochondria are double membrane-bound organelles found in almost all eukaryotes. One of their most important functions is to meet the energy needs of cells in tissues, which is accomplished largely through the oxidative phosphorylation process of aerobic respiration [20]. The oxidative phosphorylation of mitochondria involves many steps, many of which can be altered by critical illness, resulting in mild or severe pathological consequences.

Decreased mitochondrial respiratory activity is an essential part of organ dysfunction under critical conditions, especially in shock, similar to the potential effects of impaired microcirculatory perfusion and tissue oxygenation [21]. The production of ATP by mitochondria depends on a stable and adequate oxygen supply of tissue cells as electron receptors; thus, even if there is sufficient perfusion of tissue and adequate oxygen transport, mitochondrial dysfunction can lead to organ failure [22]. In other words, despite improvements in microcirculatory function, mitochondrial dysfunction and the subsequent reduction in ATP production may nevertheless lead to organ dysfunction or exacerbate organ failure. Animal experiments showed that, although the oxygen partial pressure in renal tissue cells did not change or even increase during acute kidney injury (AKI), hyperlactacidemia occurred, which is consistent with mitochondrial dysfunction and structural damage [23]. Additionally, there is clear clinical evidence that, despite improvements in microcirculatory perfusion, impaired mitochondrial respiration persists in patients after shock therapy and is associated with a worse outcome [24,25]. In the animal model of ALI, the function of mitochondria in alveolar cells was significantly reduced [26]. Mitochondria may also play a role in chronic pulmonary diseases, such as interstitial pulmonary fibrosis. Similarly, diaphragm dysfunction, which is the main risk factor for prolonged mechanical ventilation, is also associated with increased mortality in critically ill patients. Animal experiments have confirmed that mitochondrial dysfunction is an important pathological mechanism of diaphragm dysfunction [27]. Brealey et al. [25] found that sepsis can lead to severe abnormalities in mitochondria in limb muscles.

Notably, in addition to affecting the development of critical illness by regulating cellular energy metabolism, mitochondria also play key roles in many other aspects of cellular signal transduction, regulating gene expression and cellular calcium levels and activating the cell death pathway [28]. For example, by opening mitochondrial permeability transition pores and releasing cytochrome C, mitochondria can participate in programmed cell death [29]. Mitochondria have calcium transporters, suggesting that they may play a role in intracellular calcium homeostasis [30]. Additionally, mitochondria produce ROS, cellular signaling molecules involved in metabolic adaptation, apoptosis, and autophagy [31]. The functioning of mitochondria also significantly impacts organ function, which has been confirmed in many critical illnesses, including sepsis, intensive care unit-acquired skeletal muscle dysfunction, ALI, AKI, and severe immune dysfunction [28]. Therefore, critical illness can also be considered a mitochondrial disease, and the retention or improvement of mitochondrial function is associated with better outcomes.

## 4. The Critical Unit Concept

### 4.1. The Microcirculation–Mitochondria Relationship

The mutual influence and interaction between microcirculation and mitochondria are not difficult to understand. Oxygen is transported to tissue cells through microcirculation. Ischemia-induced or hypoxia-induced cell injuries that disturb the microcirculation lead to mitochondrial dysfunction, and even the heterogeneity of tissue cell function stems, to some extent, from the heterogeneity of microcirculatory perfusion [7]. Layec et al. [32] found that the energy metabolism of mitochondria in tissues from healthy subjects was mainly limited by microcirculatory perfusion. More interestingly, high altitude residents are associated with a rich microvascular network and significantly increased mitochondrial efficiency, which may indicate a very close relationship between them [33]. These findings emphasized the importance of microcirculatory perfusion in improving mitochondrial function. Correspondingly, mitochondria could participate in oxygen sensing and cell death signal pathways (apoptosis and necrosis), which may be an important target of microcirculatory dysfunction in critical illness. At the same time, damage to mitochondrial functioning can enhance oxygen transport by changing the metabolic and microcirculatory pathways [34].

The conditions of sepsis are instructive for studying the interaction between microcirculation and mitochondria in critically ill patients. Macrocirculatory failure is indeed common in sepsis patients; however, many such patients die despite resuscitation of the macrocirculation perfusion and improvement in cardiac output. What leads to the life-threatening organ dysfunction in sepsis are microcirculatory abnormalities. With the decrease of oxygen and glucose delivery in microcirculation, mitochondrial respiration is gradually difficult to maintain the production of ATP and even leads to mitochondrial function damage. This idea was confirmed in a clinical study by Fredriksson et al. [35], who found that reduced concentrations of energy-rich phosphates and increased anaerobic energy production in the leg muscles of septic patients may be responsible for the twofold decrease in mitochondrial content. However, organ dysfunction and excessive lactate production sometimes occur even when cellular oxygen transport is sufficient, suggesting that oxygen delivery alone cannot fully explain the changes in tissue metabolism caused by sepsis [36]. Changes in mitochondrial functioning induced by sepsis may be independent of changes in microcirculation; that is, such changes may play a distinct pathophysiological role in sepsis-induced multiple organ failure. Sepsis induces abnormalities in mitochondrial energy metabolism in many organs, damaging oxidative phosphorylation and ATP production. In addition, sepsis can impact mitochondrial function by altering the transport of mitochondrial calcium and mitochondrial protein expression [37].

### 4.2. The Endothelium–Mitochondria Relationship

Mitochondria and the endothelium also share a very close relationship. Mitochondria are important organelles that produce large amounts of ATP through electron transport chains [38]. Mitochondrial dysfunction increases ROS, energy stress, and cell death [39]. Ischemia and hypoxia promote mitochondrial dysfunction in the endothelium, an important factor leading to endothelial dysfunction.

The production of mitochondria-derived DAMPs (mtDAMPs) by damaged mitochondria triggers sterile inflammation [40]. These mtDAMPs include mitochondrial DNA (mtDNA), cytochrome C, and oxidized cardiolipin (oxCL) [41]. The release of mtDNA from damaged mitochondria into the cytoplasm is recognized, which triggers endothelial inflammation. Peptides and mtDNA can also increase endothelial cell permeability through both neutrophil-dependent and neutrophil-independent pathways [39]. In addition, Huang et al. [42] demonstrated that mtDNA could inhibit endothelial cell proliferation, which suggested that the regenerative capacity of endothelial cells that survived inflammatory injury was impaired. By contrast, in the mouse model of inflammatory lung injury, blocking mtDNA recognition improved the regenerative ability of endothelial cells [42]. OxCL plays a proinflammatory role by recruiting more monocytes to the intima layer of blood vessels [41]. The inhibition of mitochondrial respiration under hypoxia increases the production of mitochondrial ROS (mitoROS), which plays an important role in endothelial cells’ oxidative stress and related inflammatory responses [43]. Activation of the inflammasome pathway can also cause endothelial dysfunction. Additionally, Wang et al. [44] showed that stimulating mitochondrial fission with caffeine promoted endothelial cell migration and angiogenesis. Similarly, Yan et al. [45] found that mito-TEMPO, a mitochondria-targeted antioxidant, could improve mitochondrial function and reduce endothelial apoptosis.

**Figure 1:**
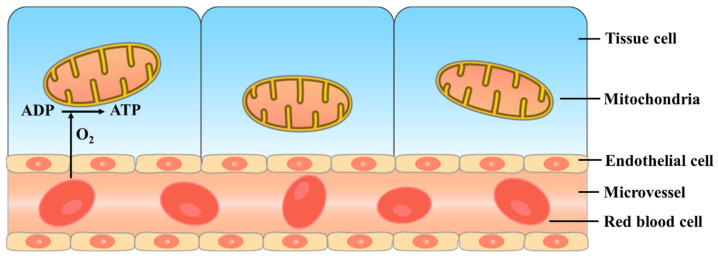
Composition of the critical unit. The critical unit mainly comprises microcirculation and mitochondria. Microcirculation includes the microvascular network (arterioles, capillary networks, and venules) and endothelial cells, especially EGC. Mitochondria, which are double membrane-bound organelles, have many important functions, one of which is to meet the energy needs of tissue cells. There is a close relationship between microcirculation and mitochondria in the occurrence and development of critical illness; thus, we combined them as the critical unit. ADP: adenosine diphosphate; ATP: adenosine triphosphate; EGC: endothelial glycocalyx.

Clearly, both microcirculation and mitochondria are vital energy-supplying apparatuses whose dysfunction is involved in critical illnesses. Because microcirculation and mitochondria mutually influence and interact in the occurrence and development of critical illness, with both playing a pivotal role, we combined them as the critical unit. Damage to the critical unit includes microcirculatory and endothelial dysfunction, as well as mitochondrial dysfunction and disrupted energy metabolism (Figure 1).

## 5. Monitoring the Critical Unit

With the ultimate goal of macrocirculatory hemodynamic optimization being to improve the critical unit, it is extremely important to monitor the critical unit itself. However, the ability to monitor the critical unit in clinical practice is relatively limited, in part because of the lack of real-time synchronous monitoring technology to assess microcirculatory perfusion and mitochondrial function. Although no high-quality randomized controlled trials have confirmed that monitoring techniques improve the prognosis of critically ill patients, effective monitoring is an important prerequisite for accurate, targeted treatment. For this reason, intensive care physicians actively seek practical monitoring technologies and promote innovations to existing devices. The emergence of several new technologies and devices is expected to solve the initial problem of critical unit monitoring (Table 1).

### 5.1. PpIX-TSLT and COMET

The balance between the supply and demand of oxygen in microcirculation is also affected by mitochondrial function, but the currently available indicators or techniques used to evaluate microcirculation, such as lactate, capillary refill time (CRT), sublingual dark-field imaging, and near-infrared spectroscopy, lack the capacity to directly assess mitochondrial function [46]. The recently developed protoporphyrin-IX delayed fluorescence lifetime technique (PpIX-TSLT) allows for the real-time noninvasive assessment of mitochondrial oxygen tension (mitoPO_2_) in the skin, which reflects mitochondrial oxygen utilization and function [56]. Wefers Bettink et al. [47] confirmed through a rat model that PpIX-TSLT could be used to indicate impaired mitochondrial oxygen utilization at the organ level, which is a more sensitive marker than lactate. In 2016, the success of PpIX-TSLT tested in a study of healthy volunteers triggered the development of the Cellular Oxygen METabolism (COMET) system (Photonics Healthcare, Utrecht, The Netherlands), suggesting its potential for clinical studies [49]. COMET is a monitoring system for repetitive noninvasive measurements of mitoPO_2_ in human skin and has been tested in clinical studies of healthy volunteers and patients in perioperative and intensive care settings [48,50,51]. Importantly, COMET can be used not only to measure mitoPO_2_ in the skin but also to evaluate mucosal oxygenation in the gastrointestinal system through endoscopy [57]. Recently, Hilderink et al. [46] established a mathematical model showing that the range of skin mitoPO_2_ was approximately 40–60 mmHg in the physiological state, further enhancing the applicability of this technique.

Skin mitoPO_2_ also reflects the oxygen supply and demand of the microcirculation to a certain extent, although there are some limitations at present [58]. As the single-parameter monitoring model is rapidly losing its effectiveness in clinical practice, multiparametric or multimodal real-time monitoring is becoming increasingly popular. Therefore, one proposal is to combine mitoPO_2_ with other microcirculation indicators, such as lactate and CRT, to reflect the condition of the critical unit. Although there remains a lack of effective means to integrate these parameters systematically, this combination may provide a feasible approach for monitoring the critical unit.

### 5.2. FMSF and FMSF-PORH

Mitochondria are the energy factories of cells. Mitochondrial nicotinamide adenine dinucleotide (NADH) is the most sensitive indicator of the supply-demand balance of oxygen. A previous study confirmed that monitoring the changes in NADH fluorescence could be utilized to reflect the functioning of mitochondrial energy metabolism in cells [55]. Katarzynska et al. [52] developed a technique called flow-mediated skin fluorescence (FMSF) to monitor the intensity of NADH fluorescence emitted from forearm skin tissue. As a new, noninvasive method for the evaluation of vascular circulation and mitochondrial metabolic regulation, FMSF can analyze dynamic changes in the emission of NADH fluorescence from skin tissue, thus providing information about mitochondrial metabolic status and intracellular oxygen transport [53]. Additionally, FMSF can be combined with the post-occlusive reactive hyperemia (PORH) test, namely FMSF-PORH, to evaluate and analyze reactive hyperemia response, hypoxia sensitivity, and the normoxia oscillatory index. FMSF-PORH can not only be utilized to diagnose macrocirculatory dysfunction, but also to evaluate the microcirculatory perfusion condition and the compensatory capacity of the microcirculation in pathological processes [53]. Through multiparametric evaluation and analysis, FMSF-PORH may realize the achievement of critical unit monitoring. Although the usefulness of this technique has been confirmed and applied in patients with diabetes, it has not been used in critically ill patients [54].

### 5.3. CritiView

Mayevsky et al. [55] developed a device called CritiView (CRV, CritiSense Ltd., Tel Aviv, Israel), which is based on the principle of monitoring changes in mitochondrial NADH fluorescence. They tested it in real time animal models and patients by measuring the following four different parameters in the urethral wall via a three-way Foley catheter connected to a multiparametric optical device: mitochondrial NADH redox state, microcirculatory blood flow, blood volume, and hemoglobin saturation. It can be utilized to evaluate the energy metabolism of tissue cells while simultaneously monitoring these four parameters, clarifying the main causes of tissue cell viability and energy disturbance. Real-time multiparameter monitoring is more advantageous than single-parameter models in a number of ways. To better monitor changes in the critical unit under abnormal perfusion conditions (e.g., ischemia or reperfusion), the supply-demand balance of oxygen and related factors of energy metabolism should be evaluated systematically. It is necessary to simultaneously assess the mitochondrial energy status, tissue oxygen saturation, and microcirculatory blood flow in both experimental and clinical environments [55]. In vitro and animal experiments have confirmed the feasibility of CRV, and similar results have been obtained in patients undergoing various cardiovascular surgeries.

With more and more experts and clinicians realizing the close connection between microcirculation and mitochondria, the integral nature of the critical unit is gaining attention and leading to the rapid development and improvement of novel technologies and devices. Such developments are encouraging and indicate that the potential of systematic, real-time monitoring of the critical unit will soon be realized. Accordingly, a new era of monitoring that is becoming ever closer to the true nature of tissue perfusion is fast approaching—the era of critical unit monitoring.

## 6. Intervention and Protection of the Critical Unit

In critical illness, there is often an obvious separation between macrocirculatory hemodynamics and microcirculatory function that typically results in a hemodynamic imbalance between macrocirculation and the microcirculation [59]. Notably, various macrocirculation-guided intervention strategies often lead to untimely treatment or over-treatment, both of which are related to the poor prognosis of patients [60]. Therefore, we emphasize the importance of critical unit-guided treatment strategies. Taking consideration of the critical unit does not equate to abandoning macrocirculatory therapy. On the contrary, the treatment priority remains the restoration and stabilization of the macrocirculation perfusion; however, the ultimate goal must be to improve the function of the critical unit. Although there are no promising microcirculation- or mitochondria-guided preclinical treatment strategies in clinical practice, it is undeniable that the concept of critical unit-guided treatment is also gradually changing the ways of clinical practice. The treatment of critical illness has gradually entered a new phase of improving microcirculatory perfusion, restoring endothelial cells, and realizing mitochondrial resuscitation [21].

### 6.1. Microcirculation-Guided Treatments

Improving microcirculatory perfusion is the core and foundation of critical unit-guided treatment. In microcirculation-guided treatments, the improvement of endothelial function and the heterogeneity of microcirculatory perfusion are two aspects that cannot be ignored. Thus, many new understandings of fluid resuscitation and drug intervention have been generated.

The resuscitation strategy in critical illness, especially in patients with shock, has been widely discussed. Fluid resuscitation is one of the most commonly used interventions in hemodynamic therapy [61]. Based on the understanding of the critical unit, the strategy of fluid resuscitation has gradually moved from targeting macrocirculation to targeting endothelial cells, which is reflected by the increasing attention of clinicians to the effects of different types of resuscitation fluids, which may aggravate or improve glycocalyx shedding, endothelial activation, and coagulation dysfunction [62]. Many experiments have demonstrated that crystalloids and gelatins may cause the degradation and shedding of the EGC; on the contrary, plasma and albumin have protective effects on vascular endothelium and microcirculation [63,64,65]. In addition, although hydroxyethyl starch may protect and restore the EGC, its use is seriously restricted by adverse events such as AKI and coagulation dysfunction [66]. The increasing emphasis on microcirculation and the endothelium has greatly impacted the choice of resuscitation fluid in clinical practice. One proposal is to increase the priority of hyperoncotic albumin solutions [62]. Although there have been no high-quality clinical studies to confirm the effects of albumin and plasma on the prognosis of critically ill patients, their potential benefits may inspire new ideas for clinical decision-making.

Various commonly used clinical drugs, such as nitroglycerin and dobutamine, have also attracted much attention. In theory, using vasodilators may improve microcirculatory perfusion by increasing the microvascular driving pressure. However, at present, there are often contradictory results in related studies. As early as 2003, Spronk et al. [67] found that intravenous nitroglycerin (0.5 mg) increased sublingual microvascular perfusion, but this conclusion was subsequently questioned. In 2010, a clinical study by Boerma et al. [68] found that nitroglycerin could not significantly improve sublingual microvascular perfusion and prognosis in septic patients. Similarly, Chen et al. [69] found that intravenous nitroglycerin (1–5 mcg/kg/min) did not improve microcirculatory perfusion in patients with hypothermic cardiopulmonary bypass during rewarming. However, in the same year, Greenwood et al. [70] found that local application of nitroglycerin could improve microcirculatory perfusion in patients after cardiac surgery.

Dobutamine also has a partial vasodilatory effect. However, due to the few studies evaluating the effect of dobutamine on microcirculation in critically ill patients and related studies also having contradictory results, whether dobutamine could improve microcirculatory perfusion needs to be confirmed by further high-quality studies [71]. In a prospective clinical study of patients with septic shock, De Backer et al. [72] demonstrated that dobutamine infusion (5 μg/kg·min) improved microcirculatory perfusion and was associated with a decrease in lactate concentration. However, in another clinical study, Hernandez et al. [73] did not find the effect of dobutamine on sublingual microcirculation or peripheral perfusion. In addition, Ospina-Tascon et al. [74] found that low-dose dobutamine may reverse the heterogeneity of microcirculatory perfusion and improve tissue O_2_ consumption in a swine model of fecal peritonitis. Recently, however, Chommeloux et al. [75] found that when the macrocirculation of patients with refractory cardiogenic shock supported by venoarterial extracorporeal membrane oxygenation recovered, increasing dobutamine (above 5 μg/kg/min) did not further improve microcirculation.

In addition, animal experiments by He et al. [76] confirmed that the administration of tetrahydrobiopterin, a nitric oxide (NO) synthase cofactor, could effectively restore sublingual microcirculation, which means that NO may play a favorable role in improving microcirculatory perfusion. Unfortunately, Trzeciak et al. [77] found that inhaled NO failed to improve microcirculatory perfusion in septic patients after macrocirculatory optimization. Iloprost, a prostaglandin I_2_ analog, could improve renal perfusion in endotoxemic rats, but clinical data are lacking [78]. Depret et al. [79] found that the intravenous iloprost could improve skin perfusion in septic shock patients. Based on this positive result, Legrand et al. [80] conducted a multicenter randomized controlled trial called I-MICRO (NCT 03788837) to provide evidence for the efficacy and safety of ilomedin, a prostacyclin analog, in adult patients with septic shock and persistent microcirculatory disorders.

It is not difficult to see that there is no strong evidence that any drug could significantly improve microcirculatory perfusion, nor have studies demonstrated the benefits of one drug over another.

### 6.2. Mitochondria-Guided Treatments

Based on an in-depth understanding of the relationship between critical illness and mitochondria, mitochondria-guided treatment strategies have also attracted much attention. Of course, it is worth noting that although several promising mitochondria-guided treatments exist, none of them have been clinically approved.

In a controlled clinical trial, Murray et al. [81] recently found that the mitochondria-targeted antioxidant, mitoquinone, improves vascular endothelial function by reducing mitoROS. Metformin treatment is beneficial for attenuating the opening of mitochondrial transition pores, stimulating mitochondrial biogenesis, and reducing mitoROS production [21]. Nevertheless, it must be used cautiously in patients with shock because of its potential for causing renal damage and serious side effects, such as hyperlactacidemia [82]. The inhibitory effect of cyclosporine A on mitochondrial permeability transition pores has shown promise in preclinical sepsis models and large animal models of traumatic brain injury [83]. Additionally, treatment with non-mitochondria-targeted antioxidants has been shown to prevent conditions that affect critically ill patients, possibly by reducing the level of mitochondrial oxidative stress. Specifically, vitamin C has been used to slow the development of vasogenic shock in sepsis [84]. Similarly, the combination of hydrocortisone, vitamin C, and thiamine in sepsis reduced mortality, the incidence of organ failure, and the dosage of vasopressor medications [85]. Melatonin may improve the production of ATP by reducing the damage to mitochondrial structure and function, thus significantly affecting inflammation [86]. In addition, Truse et al. [87] also proved that melatonin could significantly regulate gastric microcirculatory oxygenation, which may be a new aspect of the protective effect. Herminghaus et al. [88] found that indomethacin significantly affects mitochondrial function in healthy rats, which is organ-specific and concentration-dependent, and its effect should be further confirmed by in vivo studies. A recent study found that mitochondrial transplantation may be valuable in restoring organ function to critically ill patients [89]. For example, the mitochondrial transplantation of ischemic myocardial tissue has been demonstrated to increase cellular energy supply, improve the function of myocardial contractility, and reduce cell death [90].

### 6.3. Regulation of the Autonomic Nervous System (ANS)

The ANS regulates both microcirculation and mitochondria, and abnormalities in ANS functioning are often present in patients with critical illness [91]. The ANS is a part of the central nervous system, which provides unconscious control of important physiological functions and plays important roles in regulating the inflammatory response and maintaining homeostasis [91]. ANS dysfunction exists in almost all critical illnesses and leads to adverse reactions, such as myocardial injury, microvascular thrombosis, and immunosuppression; thus, regulating ANS function has become an important target of hemodynamic therapy [92]. The two efferent branches of the ANS are the sympathetic nervous system (SNS) and the parasympathetic nervous system (PNS). SNS dysfunction can lead to impaired autonomic regulation of the heart and blood vessels. In contrast, PNS dysfunction can lead to cardiac diastolic dysfunction, tachyarrhythmia, pulmonary edema, and microvascular dysfunction, ultimately resulting in hemodynamic instability [93]. Dexmedetomidine significantly inhibits the effect of the host response to infection on the SNS, thus enhancing the pressor sensitivity of norepinephrine (NE) to reduce its dosage and ultimately playing a role in stabilizing hemodynamics [94]. Morelli et al. [95] also demonstrated that esmolol increased stroke volume, reduced the dosage of NE, and even improved the 28-day mortality of patients, without significantly increasing the dosage of inotropes.

### 6.4. Importance of the Awareness of Reinjury in Critical Unit-Guided Treatment

It is well established that untimely or inappropriate intervention can cause reinjury, thus promoting the occurrence and development of critical illness, a concept that is reflected in critical unit-guided treatment. For example, fluid resuscitation remains an important strategy of critical unit-guided treatment, with current guidelines recommending crystalloids, mostly balanced crystalloids, as the first choice for fluid therapy in septic patients [96,97]. However, there are many disadvantages to using balanced crystalloids, such as EGC degradation and severe inflammation, which significantly increase vascular permeability [63]. Additionally, fluid resuscitation has potentially damaging effects, such as hemodilution, microcirculatory dysfunction, and increased risk of rebleeding [98]. Similarly, attention should be paid to the reinjury effect during the infusion of vasopressor medications, especially NE. Although NE plays an important role in hemodynamic therapy, high-dose NE can lead to intestinal and skin ischemia and can even increase mortality; thus, it is advisable to avoid the single use of high-dose NE [99]. In addition to the effects on hemodynamics, catecholamines may also have a variety of “off-target” biological effects on immunity, metabolism, and coagulation, which may have a negative impact on the prognosis of patients [100]. Catecholamines inhibit cell respiration in a dose-dependent manner. For example, the degree of mitochondrial respiratory damage and organ dysfunction directly relates to the NE infusion rate. Catecholamines could promote mitochondrial uncoupling and aggravate oxidative stress, thus promoting the progress of mitochondrial dysfunction [101]. Dexmedetomidine, esmolol, and other drugs are often used to regulate the ANS. These drugs also have reinjury effects, such as hypotension caused by decreased vascular tone or reduced cardiac output caused by inhibiting cardiac function, which may eventually lead to perfusion insufficiency [91]. In addition, NO or its derivatives may induce the uncoupling of mitochondrial respiration and inhibition of complex I and complex IV [102]. Therefore, in critical unit-guided treatment strategies, attention should also be paid to the reinjury that various treatments or interventions may cause.

## 7. Conclusions

Improving microcirculatory perfusion and mitochondrial function is a central issue following macrocirculation resuscitation, and there is growing evidence that microcirculatory and mitochondrial dysfunction are closely related to prognosis in critically ill patients. In this review, we highlighted the significant roles of microcirculation, especially endothelial cells, and mitochondria in critical illness and explained the inseparable relationship between the two. This concept of the critical unit, comprising microcirculation and mitochondria as the core, emphasizes the importance of maintaining the integrity of the critical unit to improve the status of microcirculation and mitochondria in critical illness. We also introduced several technologies and devices that function on the basis of this concept and may soon realize the achievement of critical unit monitoring. Innovations in treatment strategies that emphasize the importance of critical unit-guided monitoring and treatment are expected to further improve the concept of the critical unit. We anticipate that this review will enable more and more clinicians to understand the critical unit concept, further promoting the continuous development of critical unit monitoring and treatment.

## Figures and Tables

**Table 1:** Technologies with potential for critical unit-guided monitoring.

Technical Term	Monitoring Parameter	Author (Year) Ref.
PpIX-TSLT	mitoPO_2_	Hilderink et al. (2023) [46] Wefers Bettink et al. (2020) [47] Mik et al. (2020) [48]
COMET	mitoPO_2_	Harms et al. (2016) [49] Baumbach et al. (2019) [50] Ubbink et al. (2017) [51]
FMSF-PORH	NADH fluorescence, RHR, HS, NOI	Katarzynska et al. (2020) [52] Marcinek et al. (2023) [53] Katarzynska et al. (2019) [54]
CritiView	NADH, microcirculatory blood flow, blood volume, hemoglobin saturation	Mayevsky et al. (2011) [55]

Reference numbers are indicated by square brackets. PpIX-TSLT: protoporphyrin-IX delayed fluorescence triple state lifetime technique; mitoPO_2_: mitochondrial oxygen tension; comet: cellular oxygen METabolism; FMSF-PORH: flow-mediated skin fluorescence combined with post-occlusive reactive hyperemia; NADH: nicotinamide adenine dinucleotide; RHR: hyperemia response; HS: hypoxia sensitivity; NOI: normoxia oscillatory index.

## Data Availability

Not applicable.
